# The Management of Post-Partal Hemorrhage

**Published:** 1892-09

**Authors:** 

**Affiliations:** France


ATLANTA
MEDICAL AND SURGICAL JOURNAL.

VOL. IX. SEPTEMBER, 1892. No. 7.

EDITED BY
LUTHER B. GRANDY, M. D., AND MILLER B. HUTCHINS, M. D.,
WITH THE CO-OPERATION OF
H. V. M. MILLER, M. D., LL. D., VIRGIL O. HARDON, M. D., AND
FLOYD W. McRAE, M. D.

### *ORIGINAL COMMUNICATIONS.*

### THE MANAGEMENT OF POST-PARTAL HEMORRHAGE.

BY PROFESSOR GRYNFELT,
FRANCE.

[Translated from the *Archives de Tocologie et de Gynecologie* by Dr. L. B. Grandy, Atlanta, Ga.]

In the presence of this grave condition what, then, are we to do? There are two classes of cases: the placenta may have been already expelled, or it may be still partly adherent. If the placenta has been expelled you will be forced at once to stop the uterine inertia by such direct stimulation as uterine expression or kneading the fundus of the organ. Then, as soon as you feel that there is blood in the uterus, introduce your right hand, rendered thoroughly aseptic, into the cavity and remove the clots. The internal excitation produced by your hand will cause the uterus to contract. To these means you may then add the employment of ergot of rye, or of ergotine. * * * * *

If, in spite of these different methods, the blood continues to flow, there is yet a hæmostatic agent of the greatest value, always ready, and which you ought always to try, namely, antiseptic intra-uterine irrigations of hot water. In order to diminish the irritation which the passage of a liquid, raised to a sufficiently high temperature (113° to 118°), would provoke on the surface of the perineum, it is best to cover this region with a thin coating of vaseline. In commerce you will find different sorts of irrigators which may be easily transported. The simplest and the best is the fountain irrigator. Whenever you have not at hand carbolic acid, or corrosive sublimate, or other antiseptic material, have recourse to boiled salt-water. You will obtain from it about the same advantages. Very little pressure is necessary for these irrigations; the reservoir should be raised only a little above the level of the bed. There is one precaution which you ought to take; namely, before introducing the canula into the vagina, clear the rubber-tube of all air which it may contain. There have been sudden deaths caused by the entrance of air into the uterine sinuses and from thence into the general venous circulation.

After having passed many litres of water into the uterus, if the hemorrhage is not checked, practice compression of the abdominal aorta. This method was first carried out with the hand in the uterine cavity. In 1825 Ulsamer exerted pressure on the aorta through the abdominal wall. The happy results which he obtained did not fail to attract attention to this new method; it was not slow to find in our country many enthusiastic partisans, and it became the subject of many communications to the Academy of Medicine.

Compression of the aorta is such an excellent procedure that I cannot advocate it too strongly. Besides arresting the flow of blood very often, you are thus able more especially to prevent the conditions of syncope and cerebral anæmia which constitute two very serious accidents. It is then not too much to say, in the language of Chailly, that "the hand that compresses the aorta is the hand that holds the victim on the edge of a precipice."

To apply this compression you should depress with the fingers of one hand the abdominal wall immediately above the fundus of the uterus. You will then feel the pulsations of the aorta with more ease than you can those of the radial artery.

But your fingers will quickly become tired if you do not bring to their aid the other hand, which should be applied sufficiently firmly on top of them to maintain them in position. In fact, it is the second hand which exerts the necessary pressure, the first only acting as an intermediary.

The duration of this maneuvre is very variable. Baudelocque is said to have practiced it for four hours. In this case (patient being exhibited—*Tr.*) I maintained it for two hours, and, thanks to her, I have saved the life of my patient. It is an excellent way of saving time; in fact, it permits the ergot, previously administered, to put an end to the inertia.

Some authors have praised injections of very cold water; but as these have sometimes been followed by death from arrest of the heart, I advise you to reject them entirely.

I now come to another important method which, thanks to antisepsis, is becoming more and more used; I refer to the tampon, vaginal and intra-uterine. When Leroux, in 1775, advised for the first time stuffing the vagina with a tampon, the liveliest protests went up on every side; some said that this means was not only useless but dangerous, since it was transforming a hemorrhage external into a hemorrhage internal. This charge is far from being well-founded; in the first place, the quantity of blood which may thus collect in the cavity of the uterus is not sufficient to produce very serious consequences, and moreover, what is there excites by its presence the contractions of the uterine muscle. It is nevertheless true that the vaginal tampon, during a long time, gained only a few advocates. Négrier continued to resort to it. As for myself, I have always proscribed it.

The true intra-uterine tampon, recently devised, was first practiced by Fritsch, Skutzch and Metske in Vienna. Soon afterwards, in 1887, Duhrssen published a work tending to demonstrate the incontestable value of the new method. In France,

Auvard, in his *Obstetrical Works* which appeared in 1889, described the procedure at length, and related the excellent results which it had furnished him. The intra-uterine tampon, which must not be confounded with the vaginal tampon, is infinitely preferable, in my opinion, to the perchloride of iron and other hæmostatics of that nature. It brings about an arrest of the hemorrhage in two ways ; in determining the contraction and retraction of the uterus, and in directly obliterating the mouths of the blood-vessels. Executed antiseptically it becomes, moreover, a method absolutely harmless.

To practice it place the woman on the edge of the bed, the legs elevated and held apart by assistants. The uterus is brought down, either by seizing a lip of the cervix with forceps, or by an assistant making pressure on the fundus. If this maneuvre does not succeed insert a Sims' speculum and at the same time introduce two fingers of the left hand into the cervical canal ; these will serve as a guide along which you can carry into the uterus with long dressing forceps a strip of iodoform gauze. The outer end of the gauze should carry a thread with which it may be extracted later. If you judge that this one is not sufficient you may introduce a second band of gauze, or a third ; the organ will thus be completely filled. You should finish by placing some antiseptic tampons in the vagina. The uterine tampon is first removed after twenty-four hours, and then after forty-eight hours. I do not think that it is necessary to exceed this last interval of time. By that time we have done with our patient. This precaution seems to me necessary if we do not wish to interfere with the retraction of the organ. You may be able by this method completely to suppress a hemorrhage, even when all other means have failed. Its success has entirely dissipated certain fears which were entertained with regard to it.

Having arrested the hemorrhage it will be necessary to occupy yourself with the general condition of the woman. One should combat the tendency to syncope and chills by hypodermic injections of ether and stimulating drinks. In such cases do not be afraid to administer large doses of alçohol ; the woman will bear them well.

All these manipulations are applicable, as you will see, to cases in which the placenta has been expelled. If this is still in the uterus your first care should be to remove it by manual extraction. The act of extraction is usually followed by a cessation of both the inertia and hemorrhage.

In some cases, as with our patient here, after some days the bleeding will recommence. Then you ought to think of some foreign body retained within the uterus. Examine the organ carefully. If a tumor exists, remove it ; if there are blood clots, cause their expulsion by intra-uterine injections ; if there remains some débris of membranes, practice curettage, exercising the usual precautions.

